# Differences of the Plasma Total Lipid Fraction from Pre-Foaling to Post-Foaling Period in Donkeys

**DOI:** 10.3390/ani12030304

**Published:** 2022-01-26

**Authors:** Anastasia Lisuzzo, Francesca Bonelli, Micaela Sgorbini, Irene Nocera, Giulia Cento, Elisa Mazzotta, Luca Turini, Mina Martini, Federica Salari, Massimo Morgante, Tamara Badon, Enrico Fiore

**Affiliations:** 1 Department of Animal Medicine, Production and Health, University of Padova, Viale dell’Università 16, 35020 Legnaro, Italy; anastasia.lisuzzo@phd.unipd.it (A.L.); giulia.cento@unipd.it (G.C.); elisa.mazzotta@unipd.it (E.M.); massimo.morgante@unipd.it (M.M.); tamara.badon@unipd.it (T.B.); 2 Department of Veterinary Science, University of Pisa, Viale delle Piagge 2, 56124 Pisa, Italy; francesca.bonelli@unipi.it (F.B.); micaela.sgorbini@unipi.it (M.S.); irene.nocera@vet.unipi.it (I.N.); luca.turini@phd.unipi.it (L.T.); mina.martini@unipi.it (M.M.); federica.salari@unipi.it (F.S.)

**Keywords:** plasma fatty acids, gas chromatography, donkeys, peripartum period, metabolism

## Abstract

**Simple Summary:**

An association between increased metabolic demands and reduced dry matter intake is observed from late gestation to early lactation in donkeys. Furthermore, little is known about the nutritional and energy requirements of this period in animals. Changes in energy metabolism make donkeys more susceptible to metabolic diseases such as hyperlipemia, which is characterized by the mobilization of fatty acids from adipose tissue. A better knowledge of this period could improve animal husbandry, well-being, and health. The aim of this study was to analyze the plasma total lipid fraction, to highlight metabolic changes from the pre-foaling to post-foaling periods, using the gas chromatography technique. Our findings reveal a greater risk of metabolic disease in late gestation to early lactation in donkeys.

**Abstract:**

The period from late gestation to early lactation is characterized by changes in energy metabolism. The aim of the current study was to analyze the plasma total lipid fraction using gas chromatography (GC) analysis, in order to highlight metabolic changes from the pre-foaling to post-foaling periods. Eleven pluriparous dairy jennies (mean age of 11.88 ± 3.79 years) belonging to the Amiata donkey breed were enrolled. Blood sampling was performed at 15 days before foaling (T0), and 15 (T1), 30 (T2), 60 (T3), and 90 (T4) days after foaling, for biochemical and GC analysis. A total of 37 fatty acids were identified in plasma samples: 4 medium chain (MCFA), 24 long chain (LCFA), and 9 very-long chain (VLCFA) fatty acids. Among them, 20 fatty acids changed significantly, and two fatty acid showed a trend toward significance. Furthermore, the LCFA, saturated, unsaturated, monounsaturated, and polyunsaturated ω-3 fatty acids changed significantly during the study period. The main alterations were between T0 and the other time points and appeared to be related to lipid metabolism, cellular structure and function, and inflammatory and immune responses. Our findings reveal greater energy requirements at the end of gestation compared to early lactation in donkeys.

## 1. Introduction

Donkey products, specifically milk, are of increasing interest in European society [1]. This interest derives from their use in the prevention or treatment of cardiovascular, autoimmune, and inflammatory diseases in humans [2]. The consequence is an increased growth in the number of donkey farms [1,2]. Models to predict the nutritional and energy requirements for these animals are based on domestic horses. However, horses and donkeys are remarkably different in many aspects, and the metabolic changes in donkeys are not well understand compared to in horses [3,4,5,6]. Furthermore, few and no data are available for lactation and pregnancy requirements, respectively, in donkeys [6,7].

The period from late gestation to early lactation represents a physiologically unstable phase, due to the changes in energy metabolism for the foals’ growth, delivery, and for the beginning of lactation [8,9,10]. An association between increased metabolic demands and reduced dry matter intake, due to the lower abdominal space available for feed, is observed during this period [6,11]. This condition makes animals more susceptible to nutritional, infectious, and metabolic diseases [8,12,13]. Hyperlipemia is a metabolic disease caused by mobilization of fatty acids from adipose tissue, which frequently affects pregnant and lactating donkeys [5,12]. Therefore, particular conditions, such as pregnancy and lactation, may influence biochemical results [2,3,11]. 

Gas chromatography (GC), singularly or coupled with mass spectrometry (GC-MS), is a well-established technique for identifying and quantifying fatty acids in samples [14,15]. This technique can be used to identify the total lipid fraction, which can indicate different aspects of the metabolism that are not always identifiable by standard biochemical analysis [16]. In addition, the use of plasma as a sample type is useful, because this matrix can report changes present in all organs, without being influenced by the activity of the mammary gland, as would be in the case of milk as a sample type [16,17].

Based on all these reasons, the aim of the current study was to analyze the plasma total lipid fraction using GC analysis, to highlight metabolic changes from the pre-foaling to post-foaling period in Amiata donkeys. 

## 2. Materials and Methods

### 2.1. Animals

Eleven dairy jennies belonging to the Amiata donkey breed were enrolled. All animals were pluriparous and from 6- to 18-year-old, with a mean age of 11.88 ± 3.79 years. The study was approved by the Ethical Committee of the University of Pisa (Prot. N. 22/19). An owner’s written consent was also obtained at the beginning of the protocol. All the jennies belong to the regional stud farm ‘Le Bandite di Scarlino’ (Grosseto, Italy) and were housed at the Veterinary Teaching Hospital (VTH) ‘Mario Modenato’, Department of Veterinary Sciences, University of Pisa (Italy) from the last two months of pregnancy until one month after the delivery, undergoing the same management. Animals were housed in collective paddocks (3 animals/each) until 10–15 days before the presumptive delivery, when they were moved to individual boxes (6 m × 6 m). Jennies rejoined collective paddocks when their foals were aged 15 days. Due to their gregarious nature, the jennies were able to see and hear each other whilst in the single box. Diet was based on grass hay *ad libitum*, along with a commercial concentrate feed, according to the nutrient requirements stated by the NRC recommendations [18]. 

For each enrolled animal, body condition score (BCS) was evaluated on a scale of 1 to 5 points, following the system developed by the Donkey Sanctuary [4] before clinical examination and blood sampling. The healthy status of animals was evaluated by veterinarians of the VTH and were normal throughout the whole study period.

### 2.2. Experimental Design

This study used a time-series experimental design. Animals were evaluated 15 days before foaling and (T0), 15 (T1), 30 (T2), 60 (T3), and 90 (T4) days after foaling. The blood sampling was taken from the jugular vein and stored in tubes containing EDTA and tubes containing clot activator (FL Medical, Torreglia, Italy) at each time point. Samples were then refrigerated at 4 °C and transported at the same temperature within 1 h from sampling to the University of Pisa (Italy), where they were centrifuged at 1350 *g* in 10 min (Legend RT, Sorvall; ThermoFisher Scientific Inc., Waltham, MA, USA). Two aliquots were extracted: one aliquot of serum from tubes with clot activator was used to perform biochemical analysis, while the second aliquot of plasma from tubes with EDTA was added with pyrogallol (250 µL of extracted plasma with 5 mg of pyrogallol) and stored in a 1.5 mL capacity Eppendorf. The aliquots were sent to the University Veterinary Teaching Hospital (OVUD), Department of Animal Medicine, Production, and Health (MAPS), University of Padua (Italy), in dry ice packaging, within 24 h, for biochemical and gas chromatography analysis.

### 2.3. Blood Analysis

For serum samples, the concentrations of glucose (mg/dL), β-hydroxybutyrate (BHB; mmol/L), non-esterified fatty acids (NEFA; mEq/L), cholesterol (CHO; mg/dL), phospholipids (PHO; mg/dL), triglycerides (TRI; mg/dL), total proteins (TP; g/L), albumin (g/L), total bilirubin (mg/dL), aspartate transaminase (AST; UI/L), alanine transaminase (ALT; UI/L), γ-glutamyl-transferase (GGT; UI/L), alkaline phosphatase (ALP; UI/L), lactate dehydrogenase (LDH; UI/L), creatin-kinase (CK; UI/L), creatinine (CRE; mg/dL), reactive protein C (CRP; mg/dL), urea (UREA; mg/dL), calcium (Ca; mg/dL), chlorine (Cl; mmol/L), magnesium (Mg; mg/dL), sodium (Na; mEq/L), and potassium (K; mEq/L) were evaluated. The BHB concentration was measured using β-hydroxybutyrate enzymatic kinetics (Randox, Milan, Italy); NEFAs were assessed using the NEFA RX Monza test colorimetric method (Randox, Milan, Italy). The other serum parameters were evaluated using an automatic clinical chemistry analyzer (BT3500 Biotecnica instruments SPA, Rome, Italy). 

### 2.4. Gas Chromatographic Analysis

Gas chromatographic (GC) analysis was performed on plasma samples at the laboratory of the University of Padua. The plasma samples were first methylated by HCl-methanol and then mixed to a known concentration of internal standard (C9 and C17 triacylglycerols) to perform quantification of fatty acids. The 3N methanolic hydrochloric acid was used for the methylation process of the plasma lipids. The samples obtained were placed in an oven at 100 °C × 1 h and then neutralized with a solution of potassium carbonate (K_2_CO_3_). Finally, 37 fatty acids were obtained for each plasma sample. The fatty acid methyl esters were separated and quantified in split less mode by GC using a TRACE GC/MS (Thermo Quest, Milan, Italy) equipped with a flame ionization detector (FID) and a polar fused-silica capillary column (Capillary Column Omegawax, 30 m × 0.25 mm × 0.2 µm film). Helium was used as the carrier gas, at a flow rate of 1 mL/min. Data for plasma fatty acid were calculated in mg/dL.

### 2.5. Statistical Analysis

The statistical analysis was performed using R software ver. 4.0.3 implemented with the ‘rcmdr’ package (R Core Team, Vienna, Austria). Normal distributions of data were assessed using a Shapiro–Wilk test before any analysis. A linear mixed model (LMM) was used to evaluate differences over time in biochemical parameters and plasma total lipids. The LMM included the fixed effect of time, and the random and repeated effect of the animal and the age as covariate. A pairwise comparison was performed using the Tukey method. Furthermore, linear orthogonal contrast (LOC) and orthogonal quadratic contrast (OQC) were evaluated using the S.A.S system software (version 9.4; SAS Institute Inc., Cary, NC, USA). The hypotheses of the linear model on the residuals were graphically assessed. The accepted *p* was ≤0.05, whereas a *p* between 0.05 and 0.1 was considered as trending toward significance.

## 3. Results

Animals showed significant differences over time (Table 1) for BCS (*p* < 0.0001), NEFA (*p* = 0.001), CHO (*p* < 0.001), PHO (*p* = 0.013), TRI (*p* < 0.0001), albumin (*p* < 0.0001), total bilirubin (*p* < 0.0001), UREA (*p* = 0.019), and Cl (*p* = 0.004). Whereas TP and LDH showed a trend toward significance (*p* = 0.076 and 0.078, respectively). 

A total of 37 fatty acids were identified in plasma samples using GC (Table 2): 20 fatty acids changed significantly (*p* ≤ 0.05) from late gestation to early lactation, while two fatty acids showed a trend toward significance (0.05 < *p* ≤ 0.10). According to chain length, the GC identified four medium chain fatty acids (MCFA; between 6 and 12 carbons), 24 long chain fatty acids (LCFA; between 13 and 21 carbons), and nine very-long chain fatty acids (VLFCA; over 22 carbons) in the plasma samples. Whereas 11 saturated fatty acids (SFA), 26 unsaturated fatty acids (UFA), 10 monounsaturated fatty acids (MUFA), 16 polyunsaturated fatty acids (PUFA), 7 PUFA omega-3 (ω-3), and eight PUFA omega-6 (ω-6) were identified according to the number of double bonds. 

The LCFA, SFA, UFA, MUFA, and ω-3 concentrations showed significant changes during the study (*p* = 0.009, 0.013, 0.012, 0.002, and <0.001, respectively). In addition, the ω-6 and ω-3 ratio (ω-6:ω-3) was significantly different from pre-foaling to post-foaling period (*p* < 0.001). However, the SFA and UFA ratio (SFA:UFA) was not significantly changed over time (Table 3).

## 4. Discussion

The body fat resources are represented by BCS [7]. This parameter can be used to assess the general condition and health status of animals; a progressive reduction of body weight and BCS suggests an alteration of health status [4]. Decreased dry matter intake and increased energy requirements in late gestation and early lactation may predispose animals to fat mobilization and subsequent hyperlipidemia, particularly up to 90 days of lactation, when the greatest milk yield occurs [6,11,19]. The dairy jennies used in this study showed a reduction of BCS from 15 days pre- and post-foaling to 60- and 90-days post-foaling. A reduction of NEFA concentration was also evidenced between pre-foaling and the second and third months of lactation, whereas the total plasma fatty acids (TPFA) decreased between pre-foaling and the first month of lactation, and until the end of the study. These results may underline that the highest lipid mobilization occurred in the pre-foaling period, despite the BCS not showing significant changes. After foaling, the animals returned to a physiological state, even though they showed a progressive loss of fat resources. A similar condition can be observed in small ruminants affected by ovine pregnancy toxemia [16,20,21]. Moreover, urea concentration showed a peak around the second month of lactation. An increment of this parameter may indicate a greater energy demand due to the peak of lactation [11,22]. Associated to NEFA concentration, albumin also decreased from the weeks around foaling to the second and third months of lactation, whereas TP showed a fluctuating concentration over time. Proteins, specifically albumin, are important transporters of NEFA in the bloodstream [5]. The reduction of its concentration may have been due to the reduced need for lipid mobilization during the last part of the study. 

The CHO, PHO, and TRI values showed greater concentrations in the pre-foaling compared to post-foaling period. These lipid fractions are important constituents of the very-low density lipoprotein (VLDL), which exports TRI from the liver [23]. Their greater concentration may have been due to the higher lipid mobilization in the pre-foaling period. Furthermore, a decreased concentration of TRI and CHO after foaling may indicate their use as energy resource at the start of milking [8]. The hypothesis of a greater amount of VLDL in late gestation may also be supported by myristic acid (C14:0). In fact, a progressive increase of myristic acid was associated to an increment of apolipoprotein C III (ApoCIII), a protein present in all circulating lipoproteins. Furthermore, ApoCIII may inhibit the lipoprotein lipase reactions and enhance VLDL formation in the hepatic tissue of humans [24]. In ruminants, an increment of lipid mobilization may lead to hepatic lipidosis [25]. In donkeys, hepatic infiltration of fat results from a persistent lipolysis, with a consequent hyperlipemia. Hyperlipemia can be assessed through TRI measurement, with an upper reference limit of 248 mg/dL [26]. The TRI concentration was above this cut-off at T0. Therefore, it is possible to hypothesize a state of hepatic lipidosis. However, the plasma concentration of pentadecanoic acid (C15:0) is negatively related to hepatic steatosis in children [27]. This difference may be due to the different species. Nevertheless, the hepatic biochemical profile did not show alterations over time, suggesting perhaps an initial state of fat infiltration. The only parameter altered was total bilirubin, which showed a greater concentration in pre-foaling, probably due to pregnancy cholestasis. Similarly related to the physiology of late gestation was the highest concentration of chlorine being found in the pre-partum of dairy jennies. In fact, an increase in aldosterone was evidenced in mares during the second and third periods of pregnancy. This increase could be realized to oppose the natriuretic effect of progesterone [22].

The LCFA are mobilized by adipose tissue during the negative energy balance of ruminants. This condition is considered in many cases as a para-physiological response, to support lactation in different species. Among LCFA, the most important fatty acids are palmitic (C16:0), stearic (C18:0), oleic (C18:1 ω 9), and linoleic (C18:2 ω 6) acids [28,29]. In this study, these fatty acids stand for 84–87% of TPFA in all time points, confirming their importance in providing energy in late gestation and early lactation. Moreover, LCFA showed a greater concentration in the late gestation compared to early lactation. This finding may further suggest its mobilization from adipose tissue, due to the higher energy requirements. 

The SFA concentration was reduced from the pre-foaling to post-foaling period, probably due to the alteration in palmitic and stearic acids, which represented around 92% and 50%, respectively, of SFA at T0. However, the reduction of SFA from the pre-foaling to post-foaling period was mainly due to palmitic acid, sue to its significant reduction. Palmitic acid is an important fatty acid, related to cellular functions. In fact, it can be added to cellular protein in a process that defines palmitoylation, influencing cellular structure and the capacity to bind the lipid bilayer [30]. Palmitoleic acid (C16:1 ω 7) may derive from palmitic acid through the activity of stearoyl-CoA desaturase [31,32]. The innate immune cells, such as macrophages, contain high levels of this fatty acids and one of its isomer, hypogeic acid (C16:1 ω 9). During inflammatory events, their concentration in macrophages decrease, with a consequent release of free 16-carbons fatty acids. These fatty acids may then be used for generating compounds with anti-inflammatory effects [33].

The cis-vaccenic acid (C18:1 ω 7) can result from the elongation of palmitoleic acid. Both fatty acids showed an anti-lipogenic effect on adipocytes. Indeed, the addition of palmitoleic or cis-vaccenic acids to a culture of adipocytes reduced the lipogenesis by around 50%, contributing to the restoration of insulin sensitivity [31,34,35,36]. One compound that may also contribute to restoring insulin sensitivity is adiponectin. This protein is only secreted by adipose tissue and positively influences the β-oxidation of fatty acids in muscle, improving insulin action. Gondoic acid (C20:1 ω 9) is positively related to its concentration, probably due to an increment of both gondoic acid and adiponectin in conditions of energy deficiency [37]. The greater concentrations of these fatty acids during late gestation may suggest an attempt to manage inflammatory events and insulin resistance. Obesity is a common finding in donkeys, and it can be associated with insulin resistance [4,26]. In this study, the median value of BCS in late gestation was 4.0, indicating a high presence of fatty animals [38]. However, no significant change was observed in glycemia during the study.

A peak of concentration at one month of lactation was observed for gadoleic (C20:1 ω 11) and nervonic (C24:1 ω 9) acids, which are two very-long chain MUFA. These kinds of MUFA are associated with an increased expression of lipoprotein lipase, increasing the fatty acids oxidation and reducing the plasma free fatty acids, adipocyte size, and insulin concentration [39]. Their greater concentration may indicate a peak of energy requirement at this stage of lactation and another attempt to manage insulin sensitivity. 

The UFA concentration was reduced from pre-foaling to post-foaling period, probably due to the alteration in MUFA concentration. The fatty acid that influenced MUFA concentration more was oleic acid (C18:1 ω 9), which represented around 72% of the MUFA concentration at T0. Oleic acid is one of the fatty acids most mobilized during the negative energy balance of dairy cows, probably due to its high concentrations in adipocytes [28,40]. This fatty acid has also been found at high levels in mares, thus influencing its concentration in the reproductive system and the fertility of the animal [41]. The increase of oleic acid concentrations in late gestation may further indicate greater energy demands at this physiological stage. In addition, this could affect the cell membrane composition and fertility of the animal. Chemically similar oleic acid is heptadecenoic acid (C17:1 ω 7), which should not naturally be present in mammalian tissues [42]. In contrast, this fatty acid was found in ruminant plasma and probably reflects de novo synthesis of fatty acids in the rumen [43]. In this study, heptadecenoic acid only increased at one month after foaling (T2). Considering that some ruminal microorganisms are like those found in the large intestine of donkeys [44], it is possible that at this time point an alteration of intestinal motility and/or fermentation took place, leading to an increase in this fatty acid.

The ω-3 and ω-6 PUFA are incorporated into cellular membranes as phospholipids. An inflammatory cascade begins when these fatty acids, especially arachidonic acid (C20:4 ω 6; AA), are released from phospholipids and used to produce eicosanoids [45]. Specifically, ω-3 is involved in the production of anti-inflammatory products, such as lipoxins, resolvins, and protectins, whereas ω-6 is involved in both pro- and anti-inflammatory responses [16]. The ω-3 fatty acids derive from essential fatty acids, the α-linolenic acid (C18:3 ω 3; ALA) [46,47]. Among them, there are the eicosatrienoic (C20:3 ω 3; ETE), eicosapentaenoic (C20:5 ω 3; EPA), docosapentaenoic (C22:5 ω 3; DPA), and docosahexaenoic (C22:6 ω 3; DHA) acids. While ω-6 fatty acids are produced by γ-linolenic (C18:3 ω 6; GLA) acid, and include eicosadienoic (C20:2 ω 6), dihomo-γ-linolenic (C20:3 ω 6; DGLA), AA, and adrenic (C22:4 ω 6; ADA) acids [48,49]. All these fatty acids, except for GLA, showed a greater concentration in the pre-foaling period compared to post-foaling. The greater concentration of these ω-3 fatty acids led to an increment of total ω-3 concentration at the same time point, but this did not occur for the total concentration of ω-6. The consequence was a reduction in ω-6:ω-3 ratio at T0 compared to the other time points. These findings suggest an inflammatory status in donkeys at the end of gestation in agreement with the study of Bazzano et al. [50]. In fact, the tissue damage during the peripartum period may lead to an inflammatory response. Furthermore, the progressive increment of metabolic activity during pregnancy may induce an oxidative stress status in animals, linked to inflammation [51]. The ADA is a ω-6 that can further predispose animals to oxidative stress though an influence on antioxidant system expression, such as superoxide dismutase (SOD) and glutathione peroxidase (Gpx) [52,53,54]. The nutrients and energy may have been redirected by the inflammatory and oxidative state [29], further predisposing donkeys to fat mobilization.

## 5. Conclusions

In our study, plasma fatty acid profile was described from the pre-foaling to post-foaling period in dairy donkeys using GC analysis. The changes thus highlighted appeared to be related to (i) lipid metabolism, (ii) cellular structure and function, and (iii) inflammatory and immune responses. In particular, the animals in our study seem to show the greatest alterations in the last period of gestation, highlighting how little is still known about the energy requirements of donkeys in this productive phase. These findings confirmed the applicability of GC as a useful diagnostic tool for evaluating animal metabolism. Further studies would be necessary to explore this metabolically stressful period and to understand how to compensate for any energy deficiencies that may occur, even using GC.

## Figures and Tables

**Table 1:** Least square means and standard error of the mean (SEM) of BCS and biochemical parameters from pre-foaling (T0: 15 days before foaling) to post-foaling (T1: 15, T2: 30, T3: 60, T4: 90 days after foaling) of donkeys.

Parameters	T0	T1	T2	T3	T4	SEM	*p*	LOC ^22^	OQC ^23^
BCS ^1,^*	3.68 ^a^ 4.00 (3.50–4.00)	3.56 ^a^ 3.88 (3.19–4.00)	3.38 ^ab^ 3.50 (3.25–3.50)	3.20 ^bc^ 3.50 (2.75–3.50)	3.00 ^c^ 3.00 (2.75–3.25)	0.14	<0.0001	<0.0001	NS ^21^
Glycemia, mg/dL	70.50	61.70	65.10	62.50	50.40	8.13	NS ^21^	NS ^21^	NS ^21^
BHB ^2^, mmol/L	0.32	0.44	0.40	0.36	0.34	0.06	NS ^21^	NS ^21^	NS ^21^
NEFA ^3^, mEq/L	0.72 ^a^	0.54 ^ab^	0.45 ^ab^	0.16 ^b^	0.14 ^b^	0.11	0.001	<0.001	NS ^21^
CHO ^4^, mg/dL	109.20 ^a^	92.30 ^ab^	73.20 ^b^	68.70 ^b^	71.40 ^b^	9.91	<0.001	<0.0001	0.005
PHO ^5^, mg/dL	4.08 ^a^	3.50 ^ab^	3.26 ^b^	3.08 ^b^	3.38 ^ab^	0.19	0.013	0.009	0.016
TRI ^6^, mg/dL	277.70 ^a^	40.00 ^b^	19.50 ^b^	27.00 ^b^	36.80 ^b^	31.58	<0.0001	<0.0001	<0.0001
TP ^7^, g/L	89.40	84.90	85.30	83.20	86.60	2.48	0.076	NS ^21^	0.051
Albumin, g/L	36.10 ^a^	36.00 ^a^	33.00 ^ab^	31.70 ^b^	29.90 ^b^	1.07	<0.0001	<0.0001	NS ^21^
Total Bilirubin, mg/dL	0.60 ^a^	0.25 ^b^	0.21 ^b^	0.16 ^b^	0.20 ^b^	0.06	<0.0001	<0.0001	0.002
AST ^8^, UI/L	186.00	198.00	212.00	202.00	214.00	14.16	NS ^21^	NS ^21^	NS ^21^
ALT ^9^, UI/L	5.69	6.61	4.45	4.51	5.02	1.19	NS ^21^	NS ^21^	NS ^21^
GGT ^10^, UI/L	41.10	52.50	49.50	36.90	45.70	7.86	NS ^21^	NS ^21^	NS ^21^
ALP ^11^, U/L	461.00	473.00	556.00	530.00	545.00	91.40	NS ^21^	NS ^21^	NS ^21^
LDH ^12^, U/L	297.00	348.00	319.00	359.00	414.00	38.42	0.078	0.052	NS ^21^
CK ^13^, U/L	167.00	112.00	144.00	150.00	159.00	27.98	NS ^21^	NS ^21^	NS ^21^
CRE ^14^, mg/dL	1.22	1.36	1.23	1.11	1.11	0.08	NS ^21^	NS ^21^	NS ^21^
CRP ^15^, mg/dL	0.30	0.57	0.54	0.58	0.55	0.12	NS ^21^	NS ^21^	NS ^21^
Urea, mg/dL	33.60 ^ab^	28.90 ^a^	31.70 ^ab^	39.60 ^b^	37.30 ^ab^	2.49	0.019	0.029	NS ^21^
Ca ^16^, mg/dL	13.70	12.90	13.20	13.00	13.20	0.53	NS ^21^	NS ^21^	NS ^21^
Cl ^17^, mmol/L	115.00 ^a^	111.00 ^ab^	109.00 ^b^	108.00 ^b^	108.00 ^b^	2.67	0.004	0.015	0.029
Mg ^18^, mg/dL	2.35	2.43	2.49	2.39	2.46	0.13	NS ^21^	NS ^21^	NS ^21^
Na ^19^, mEq/L	143.00	146.00	142.00	144.00	144.00	1.94	NS ^21^	NS ^21^	NS ^21^
K ^20^, mEq/L	7.44	7.81	4.54	8.51	6.94	2.31	NS ^21^	NS ^21^	NS ^21^

^1^ Body condition score; * Least square means, median (1°–3° quartile); ^2^ β-Hydroxybutyrate; ^3^ Non-esterified fatty acid; ^4^ Cholesterol; ^5^ Phospholipid; ^6^ Triglycerides; ^7^ Total protein; ^8^ Aspartate Transaminase; ^9^ Alanine Transaminase; ^10^ γ-Glutamyl-transferase; ^11^ Alkaline phosphatase; ^12^ Lactate dehydrogenase; ^13^ Creatin-kinase; ^14^ Creatinine; ^15^ Reactive protein C; ^16^ Calcium; ^17^ Chlorine; ^18^ Magnesium; ^19^ Sodium; ^20^ Potassium; ^21^ Non-significant; ^22^ Linear orthogonal contrast; ^23^ Orthogonal quadratic contrast; ^a–c^ Mean values in the same row that differ significantly.

**Table 2:** Least square means and standard error of the mean (SEM) of identified plasma fatty acids (mg/dL) from pre-foaling (T0: 15 days before foaling) to post-foaling (T1: 15, T2: 30, T3: 60, T4: 90 days after foaling) of donkeys.

Fatty Acids	Nomenclature	T0	T1	T2	T3	T4	SEM	*p*	LOC ^3^	OQC ^4^
C6:0	Caproic acid	4.93	17.47	16.52	17.27	18.00	2.89	NS ^2^	NS ^2^	0.085
C8:0	Caprylic acid	1.89	2.07	1.96	2.00	1.73	0.70	NS ^2^	NS ^2^	NS ^2^
C10:0	Capric acid	1.03	0.66	0.91	0.58	0.66	0.16	NS ^2^	NS ^2^	NS ^2^
C12:0	Lauric acid	0.74	0.34	0.67	0.14	0.15	0.32	NS ^2^	NS ^2^	NS ^2^
C14:0	Myristic acid	3.78 ^a^	0.78 ^ab^	0.76 ^b^	0.60 ^b^	0.77 ^b^	0.71	0.021	0.018	NS ^2^
C14:1 ω 5	Myristelaidic acid	0.25	0.07	0.03	0.06	0.05	0.06	NS ^2^	NS ^2^	NS ^2^
C15:0	Pentadecanoic acid	0.76 ^a^	0.23 ^b^	0.26 ^b^	0.31 ^b^	0.40 ^ab^	0.11	0.015	0.100	0.004
C16:0	Palmitic acid	98.60 ^a^	34.30 ^ab^	26.80 ^b^	27.00 ^b^	30.70 ^b^	15.68	0.009	0.020	0.010
C16:1 ω 9	Hypogeic acid	3.33 ^a^	0.65 ^b^	0.53 ^b^	0.52 ^b^	0.70 ^b^	0.60	0.012	0.020	0.010
C16:1 ω 7	Palmitoleic acid	16.24 ^a^	4.29 ^ab^	2.62 ^b^	2.02 ^b^	1.70 ^b^	3.10	0.015	0.009	0.032
C16:3 ω 4	Hexadecatrienoic acid	0.50 ^a^	0.16 ^b^	0.17 ^b^	0.21 ^b^	0.33 ^ab^	0.05	0.003	0.094	0.0001
C17:1 ω 7	Heptadecenoic acid	0.08 ^a^	0.05 ^a^	0.45 ^b^	0.08 ^a^	0.10 ^a^	0.04	<0.001	NS ^2^	0.001
C18:0	Stearic acid	53.00	44.40	36.50	37.60	37.30	9.12	0.098	NS ^2^	NS ^2^
C18:1 ω 9	Oleic acid	74.70 ^a^	23.10 ^b^	19.00 ^b^	20.20 ^b^	25.20 ^b^	10.98	0.003	0.019	0.003
C18:1 ω 7	cis-Vaccenic acid	6.62 ^a^	2.95 ^b^	2.17 ^b^	2.11 ^b^	2.01 ^b^	0.90	0.001	0.006	0.012
C18:2 ω 6	Linoleic acid	106.20	100.60	84.00	86.30	91.80	20.86	NS ^2^	NS ^2^	NS ^2^
C18:3 ω 6	γ-Linolenic acid (GLA)	0.84	0.72	1.20	0.93	1.26	0.18	NS ^2^	NS ^2^	NS ^2^
C18:3 ω 3	α-Linolenic acid (ALA)	13.11 ^a^	2.63 ^b^	1.66 ^b^	2.16 ^b^	2.20 ^b^	1.79	<0.001	0.002	0.001
C18:4 ω 3	Stearidonic acid (SDA)	0.12	0.32	0.20	0.18	0.22	0.09	NS ^2^	NS ^2^	NS ^2^
C20:0	Arachidic acid	0.56	2.50	3.30	5.19	2.19	1.79	NS ^2^	NS ^2^	NS ^2^
C20:1 ω 11	Gadoleic acid	0.67 ^a^	2.53 ^ab^	4.41 ^b^	2.30 ^ab^	1.53 ^ab^	1.23	0.014	NS ^2^	0.005
C20:1 ω 9	Gondoic acid	0.92 ^a^	0.43 ^b^	0.49 ^b^	0.60 ^ab^	0.66 ^ab^	0.15	0.017	NS ^2^	0.004
C20:2 ω 6	Eicosadienoic acid	0.49 ^a^	0.24 ^b^	0.28 ^b^	0.35 ^ab^	0.45 ^ab^	0.08	0.011	NS ^2^	0.001
C20:3 ω 6	Dihomo-γ-linolenic acid	0.72 ^a^	0.41 ^b^	0.29 ^b^	0.29 ^b^	0.28 ^b^	0.08	<0.001	0.001	0.001
C20:4 ω 6	Arachidonic acid (AA)	2.07 ^a^	1.98 ^ab^	1.64 ^ab^	1.57 ^ab^	1.41 ^b^	0.32	0.027	0.057	NS ^2^
C20:3 ω 3	Eicosatrienoic acid (ETE)	0.39 ^a^	0.11 ^b^	0.09 ^b^	0.12 ^b^	0.10 ^b^	0.05	0.001	0.006	0.001
C20:4 ω 3	Eicosatetranoic acid	0.10	0.08	0.04	0.05	0.05	0.02	NS ^2^	0.075	NS ^2^
C20:5 ω 3	Eicosapentaenoic acid	0.88 ^a^	0.66 ^ab^	0.56 ^b^	0.54 ^b^	0.66 ^ab^	0.12	0.042	NS ^2^	0.025
C22:0	Behenic acid	0.57	0.55	0.47	0.56	0.55	0.07	NS ^2^	NS ^2^	NS ^2^
C22:1 ω 9	Erucic acid	0.06	0.04	0.05	0.04	0.05	0.01	NS ^2^	NS ^2^	NS ^2^
C22:2 ω 6	Docosadienoic acid	0.08	0.04	0.05	0.06	0.07	0.02	NS ^2^	NS ^2^	NS ^2^
C22:4 ω 6	Adrenic acid (ADA)	0.29 ^a^	0.21 ^ab^	0.21 ^ab^	0.21 ^ab^	0.19 ^b^	0.05	0.028	0.086	0.075
C22:5 ω 6	Docopentaenoic acid	0.03	0.24	0.23	0.26	0.26	0.04	NS ^2^	NS ^2^	NS ^2^
C22:5 ω 3	Decosapentaenoic acid	0.49 ^a^	0.25 ^ab^	0.18 ^b^	0.20 ^b^	0.17 ^b^	0.06	0.002	0.006	0.016
C22:6 ω 3	Docosahexaenoic acid	0.44 ^a^	0.22 ^ab^	0.18 ^b^	0.20 ^b^	0.19 ^b^	0.06	0.020	0.015	0.034
C24:0	Lignoceric acid	0.78	0.08	0.94	0.94	0.96	0.20	NS ^2^	NS ^2^	NS ^2^
C24:1 ω 9	Nervonic acid	1.89	2.53	2.61	2.44	2.16	0.52	0.072	NS ^2^	0.009
TPFA ^1^		391.00 ^a^	233.00 ^ab^	198.00 ^b^	203.00 ^b^	212.00 ^b^	61.06	0.024	0.072	0.022

^1^ Total plasma fatty acids; ^2^ Non-significant; ^3^ Linear orthogonal contrast; ^4^ Orthogonal quadratic contrast; ^a,b^ Mean values in the same row that differ significantly.

**Table 3:** Least square means and standard error of the mean (SEM) of plasma fatty acids (mg/dL) according to the length of the chain and the number of double bonds at 15 days before foaling (T0), and 15 (T1), 30 (T2), 60 (T3), and 90 (T4) days after foaling.

Parameters	T0	T1	T2	T3	T4	SEM	*p*	LOC ^9^	OQC ^10^
MCFA ^1^	2.69	8.57	6.59	7.30	5.91	2.73	NS ^8^	NS ^8^	0.067
LCFA ^2^	383.00 ^a^	220.00 ^ab^	187.00 ^b^	191.00 ^b^	202.00 ^b^	58.82	0.009	0.063	0.018
VLCFA ^3^	4.71	4.72	4.78	4.70	4.44	0.91	NS ^8^	NS ^8^	NS ^8^
SFA ^4^	160.70 ^a^	90.30 ^ab^	75.60 ^b^	79.40 ^b^	78.80 ^b^	25.48	0.013	0.060	0.026
UFA ^5^	230.00 ^a^	142.00 ^ab^	122.00 ^b^	123.00 ^b^	133.00 ^ab^	35.82	0.012	0.084	0.021
SFA:UFA	0.66	0.63	0.62	0.65	0.60	0.02	NS ^8^	NS ^8^	NS ^8^
MUFA ^6^	103.60 ^a^	35.80 ^b^	31.60 ^b^	30.10 ^b^	33.90 ^b^	16.16	0.002	0.023	0.007
PUFA ^7^	126.50	108.00	90.70	93.10	99.30	22.44	NS ^8^	NS ^8^	NS ^8^
PUFA ω-3 (ω-3)	15.57 ^a^	4.13 ^b^	2.82 ^b^	3.36 ^b^	3.50 ^b^	2.00	<0.001	0.002	0.001
PUFA ω-6 (ω-6)	110.90	104.20	87.80	89.70	95.60	21.40	NS ^8^	NS ^8^	NS ^8^
ω-6:ω-3	8.98 ^a^	24.62 ^b^	30.48 ^b^	25.52 ^b^	29.33 ^b^	3.49	<0.001	0.001	0.001

^1^ Medium chain fatty acid; ^2^ Long chain fatty acid; ^3^ Very long chain fatty acid; ^4^ Saturated fatty acid; ^5^ Unsaturated fatty acid; ^6^ Monounsaturated fatty acid; ^7^ Polyunsaturated fatty acid; ^8^ Non-significant; ^9^ Linear orthogonal contrast; ^10^ Orthogonal quadratic contrast; ^a,b^ Mean values in the same row that differ significantly.

## Data Availability

The data are available by sending an email to the corresponding author.
